# Supplementary material for: IFNβ-dependent increases in STAT1, STAT2, and IRF9 mediate resistance to viruses and DNA damage
Source: EMBO J. 2013 Sep 24;32(20):2751–63. doi: 10.1038/emboj.2013.203 (PMC3801437; doi:10.1038/emboj.2013.203)
Supplement: Supplementry Information [file emboj2013203s1.pdf]

Supplementary Information

IFNβ-dependent Increases in STAT1, STAT2, and IRF9 Mediate Resistance to Viruses and DNA Damage

HyeonJoo Cheon, Elise G. Holvey-Bates, John W. Schoggins, Samuel Forster, Paul Hertzog, Naoko Imanaka, Charles M. Rice, Mark W. Jackson, Damian J. Junk, and George R. Stark

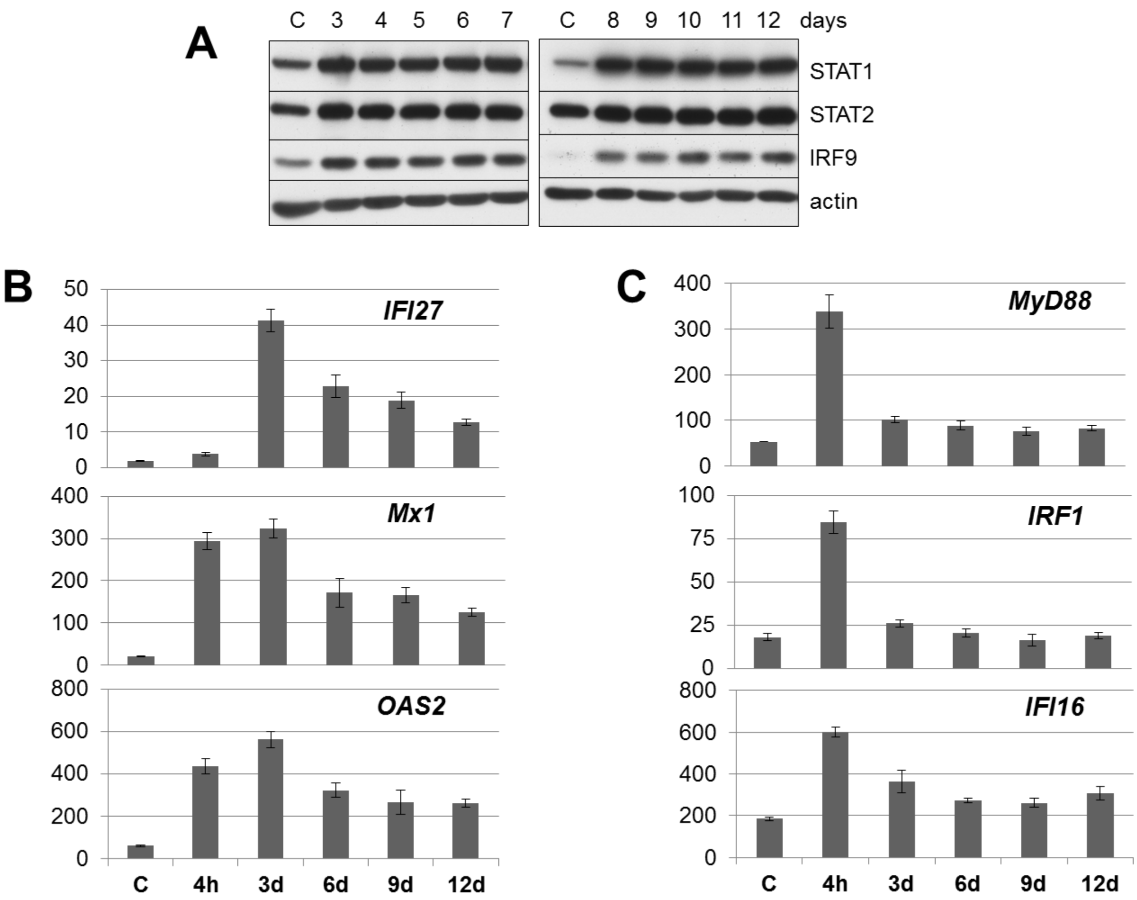

Figure S1. The expression of U-STAT1-induced antiviral genes is sustained for 12 days after IFN stimulation, along with increased levels of the STAT1, STAT2, and IRF9 proteins

(A) The levels of IRF9 and STATs 1 and 2 proteins were analyzed by the Western method in BJ normal fibroblasts collected 0-12 days after a single treatment with IFNβ (50 IU/ml).

(B and C) The expression of the U-STAT1-target antiviral genes *IFI27*, *MX1*, and *OAS2* (B) and other ISGs *MYD88, IRF1,* and *IFI16* (C) was analyzed by real-time PCR in BJ normal fibroblasts collected 0-12 days after a single treatment with IFNβ (50 IU/ml). The levels of gene expression were calculated semi-quantitatively, by using the ΔΔCt method. The data are represented as means of triplicate PCR analyses ± standard deviations (SD).

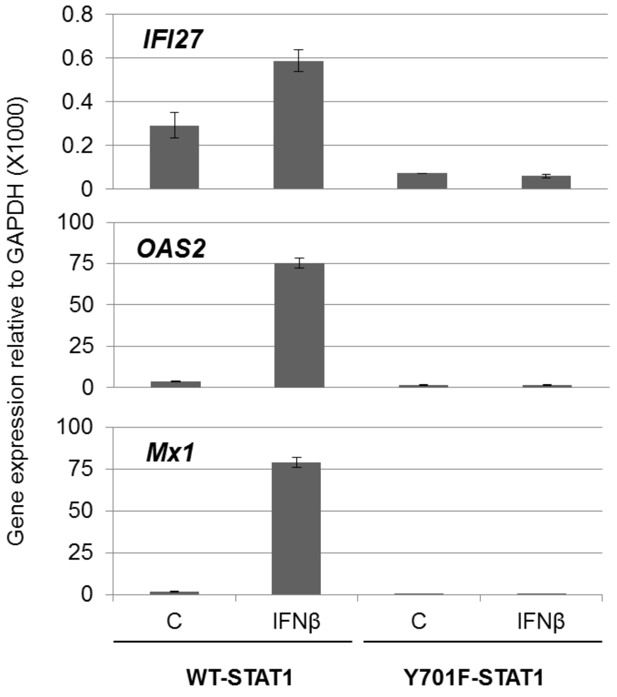

Figure S2. ISGs were not induced by IFNβ in STAT1-null fibroblasts reconstituted with Y701F STAT1

STAT1-null fibroblasts were transfected with wild-type-STAT1 or Y701F-STAT1 in the lentiviral vector, and the cells were treated with 50 IU/ml of IFNβ for 4 h. The expression of *IFI27, OAS2*, and *MX1* was analyzed by real-time PCR in untreated (C) and treated cells. The levels of gene expression were calculated semi-quantitatively, by using the ΔΔCt method. The data are represented as means of triplicate PCR analyses ± standard deviations (SD).

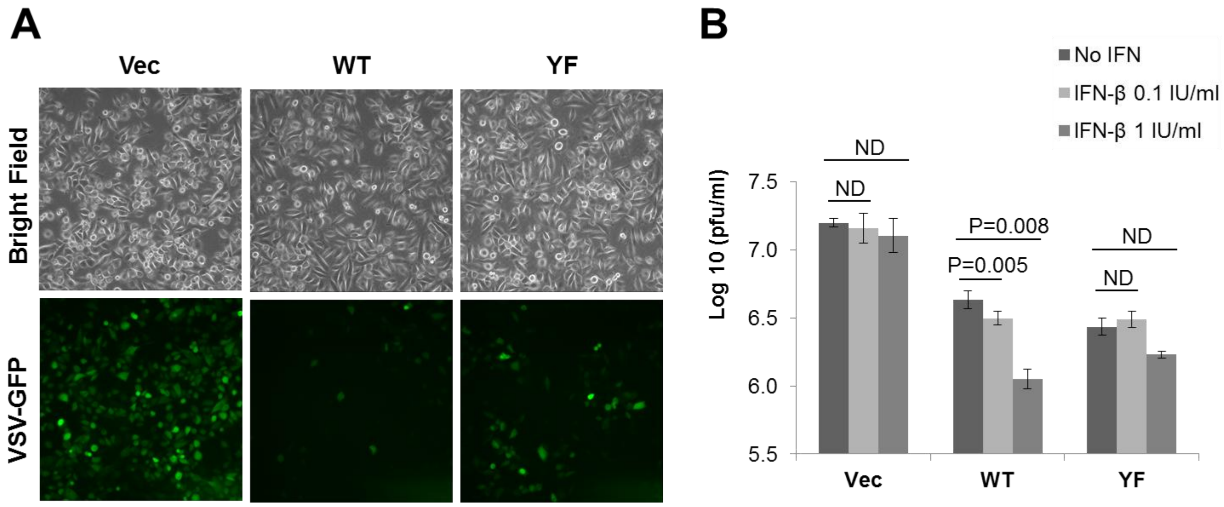

Figure S3. Antiviral effects resulting from high levels of Y701F-STAT1, STAT2, and IRF9 are not influenced by IFNβ in the media

(A) hTERT-HME1 cells expressing high levels of wild-type-STAT1/STAT2/IRF9 (WT) or Y701F-STAT1/ STAT2/IRF9 (YF) were infected with 1 MOI of GFP-expressing VSV. The GFP signal was detected by using fluorescence microscopy (X 100) 8 h after infection.

(B) hTERT-HME1 cells stably transfected with wild-type-STAT1/STAT2/IRF9 (WT) or Y701F-STAT1, STAT2, and IRF9 (YF) were pre-treated with 0.1 or 1 IU/ml of IFNβ for 18 h and then infected with 1 MOI of VSV. The cells were collected 10 h after infection and titers of infectious virus were measured by plaque assays. The data are represented as means of triplicate experiments with virus infected cells ± SD. ND, not different statistically ($P>0.05$, by two-tailed t-test). Virus titers in IFNβ-treated WT cells were significantly different compared to untreated WT cells ($P<0.01$, by two-tailed t-test).

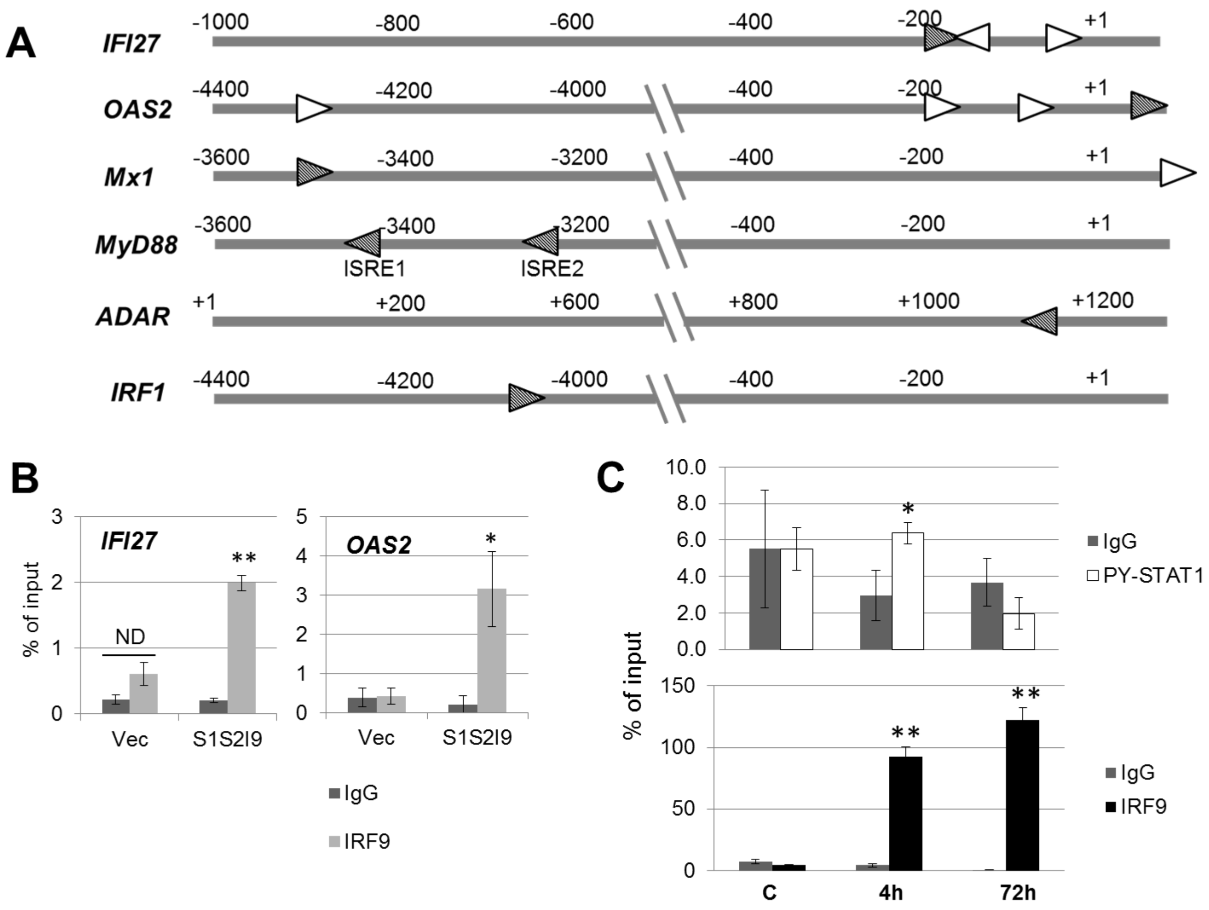

Figure S4. High levels of U-STAT1, U-STAT2, and IRF9 proteins increase the binding of U-ISGF3 to ISREs of target genes

(A) ISREs in the promoters of U-ISGF3-induced genes (*IFI27, OAS2,* and *MX1*) and ISGs not induced by U-ISGF3 (*MYD88, ADAR,* and *IRF1*) were identified in 5'UTRs and -5000 bp upstream from transcription start sites (+1) by using the transcription factor search program TFSEARCH (http://www.cbrc.jp/research/db/TFSEARCH.html, Threshold=80.0). Triangles represent ISREs and their directions (►, forward; ◄, reverse). The striped triangles are the most conserved sequences and the binding of U-ISGF3 to them is presented in Figure 4.

(B) hTERT-HME1 cells stably transfected with empty vector (Vec) or STAT1/STAT2/IRF9 (S1S2I9) were used. Cells were cross-linked with 1% formaldehyde and the cell lysates were cross-linked with DTBP. Chromatin was sheared into <1 kb lengths by sonication. Rabbit polyclonal antibody against IRF9 or comparable rabbit IgG was used for immuno-precipitations. Real-time PCR was performed to amplify the precipitated DNAs with primer pairs spanning ISREs in the promoters of the *IFI27* and *OAS2* genes. The levels of gene expression were calculated semi-quantitatively, by using the ΔΔCt method. The values represent the percentages of DNA amount in ChIP-ed samples compared to 2% input DNA. The data are represented as means of triplicate PCR analyses ± standard deviations (SD). ** represents $p<0.01$ and * represents $p<0.05$, by two-tailed t-test, compared to the IgG-control. ND, not different statistically ($P>0.05$, by two-tailed t-test).

(C) hTERT-HME1 cells were treated with 3 IU/ml of IFNβ for 4 or 72 h. ChIP assays were carried out as described above using rabbit polyclonal antibodies against IRF9 or PY-STAT1. Real-time PCR was performed as described above using primer pairs spanning ISRE of the *IFI27* gene.

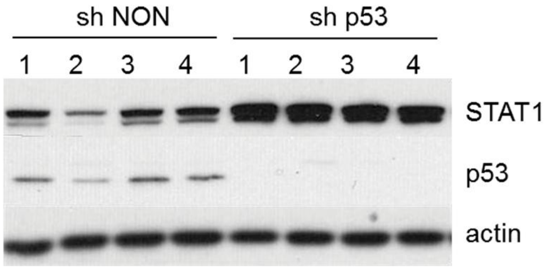

Figure S5. Knocking p53 down increases the expression of STAT1 protein

Human primary mammary epithelial cells were transfected with an shRNA against p53 (sh p53) or an unrelated shRNA (sh NON), and the expression of STAT1 was examined in individual clones, 1-4, by the Western method.

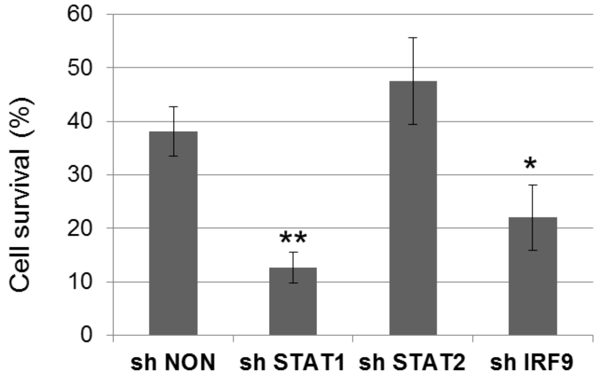

Figure S6. Knocking STAT1 and IRF9 down makes H196, a resistant SCLC line, more sensitive to DNA damage induced by doxorubicin

The resistant small cell lung carcinoma cell line H196 was transfected with shRNAs against STAT1, STAT2, and IRF9, or a non-targeted shRNA (sh NON). The cells were treated with 1 μM doxorubicin and their viability was assessed after 72 h by using an Alamar blue assay. Cell numbers were determined by generating standard curves with known numbers of untreated sh NON cells. The values are percentages of the numbers of surviving doxorubicin-treated cells compared to untreated cells (100%). The data are represented as means of triplicate of Alamar blue assays ± SD. ** represents $p<0.01$ and * represents $p<0.05$, by two-tailed t-test, compared to doxorubicin-treated sh NON cells.

Table S1. Genes induced by ISGF3, but not by U-ISGF3

The 48 genes (51 probes) were selected as the genes induced only by IFNβ, not by IFNγ or Y701F-STAT1 overexpression. The BJ fibroblasts were treated by IFNβ (3 IU/ml for 6 h), and microarray data were analyzed as described in “Materials and methods”.

PROBE_ID	SYMBOL	PROBE_ID	SYMBOL
ILMN_1734652	ARHGAP27	ILMN_1697817	PANX1
ILMN_1787595	B3GALT3	ILMN_1663927	PARP10
ILMN_1726840	B3GALT3	ILMN_1788059	PCGF5
ILMN_1669140	BLZF1	ILMN_1736548	PHACTR4
ILMN_1732278	C1orf80	ILMN_1815134	PI4K2B
ILMN_1791980	CUTA	ILMN_1755173	PLEKHA4
ILMN_1728478	CXCL16	ILMN_1810608	PNPT1
ILMN_1697864	CXorf38	ILMN_1718222	PPM1K
ILMN_1743373	DLL1	ILMN_1656953	PRKD2
ILMN_1720083	EHD4	ILMN_1728009	PRP2
ILMN_1706502	EIF2AK2	ILMN_1672398	RAB20
ILMN_1740466	FAM46A	ILMN_1727045	RASGRP3
ILMN_1750400	FLJ11286	ILMN_1813430	RNF36
ILMN_1653466	HES4	ILMN_1811498	RP3-473B4.1
ILMN_1762861	HLA-F	ILMN_1668358	SAMD9
ILMN_1803457	IFI16	ILMN_1705803	SOX9
ILMN_1686989	INSIG1	ILMN_1784364	STARD5
ILMN_1696434	LAMA1	ILMN_1689977	TDRD7
ILMN_1682336	MASTL	ILMN_1797236	TGM2
ILMN_1769550	MGC19764	ILMN_1785732	TNFAIP6
ILMN_1657435	MT1M	ILMN_1737599	TRIM5
ILMN_1738523	MYD88	ILMN_1704972	TRIM5
ILMN_1687768	NCOA7	ILMN_1797359	TRIM5
ILMN_1724898	NFE2L3	ILMN_1794612	UBE1L
ILMN_1678745	NUDCD1	ILMN_1654812	UNC93B1
ILMN_1728224	OGFR	ILMN_1798061	ZFYVE26
