# Supplementary figures and images for: IFNβ-dependent increases in STAT1, STAT2, and IRF9 mediate resistance to viruses and DNA damage
Source: EMBO J. 2013 Sep 24;32(20):2751–63. doi: 10.1038/emboj.2013.203 (PMC3801437; doi:10.1038/emboj.2013.203)

Cheon et al. Source Data  
Figure 1B

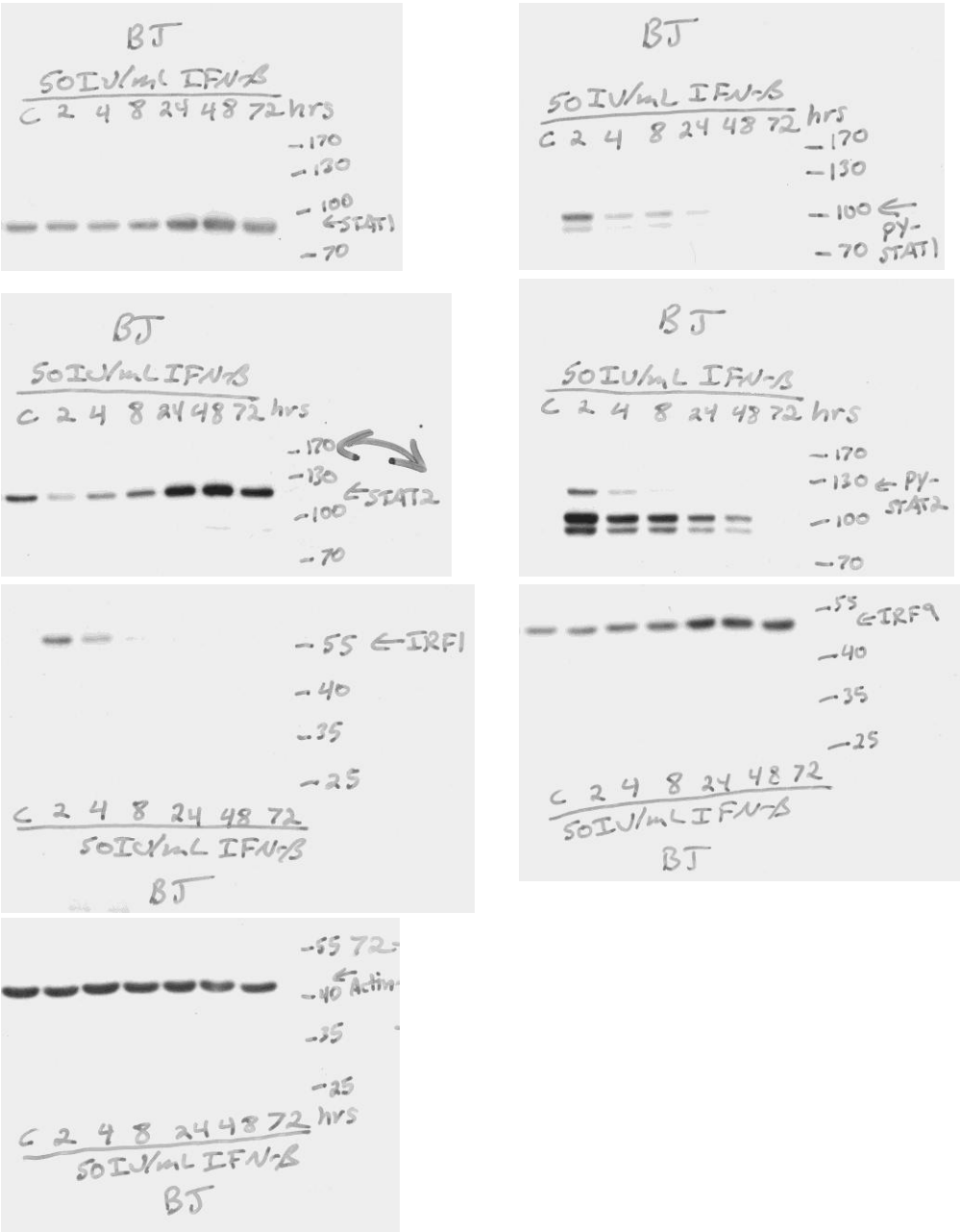

Supplement: Source Data for Figure 1B [file emboj2013203df1b.pdf]

Cheon *et al.* Source Data  
Figure 2A

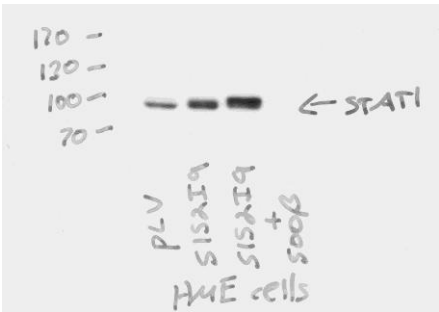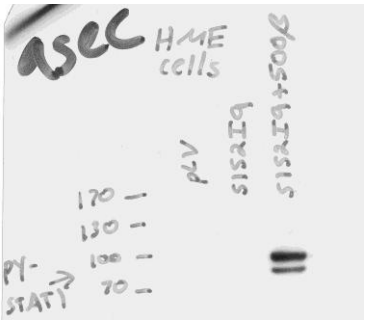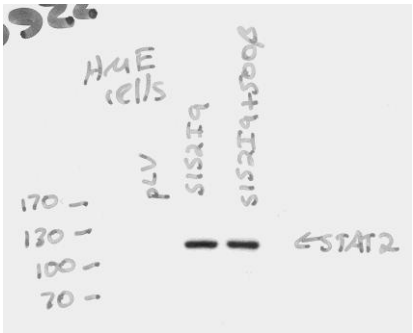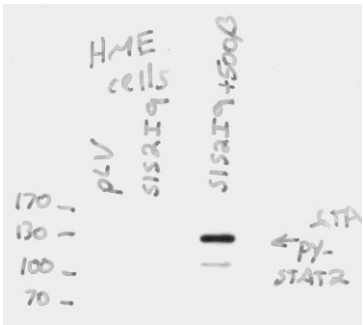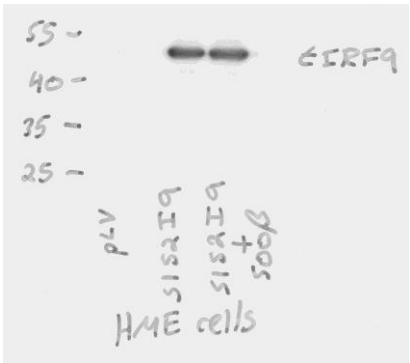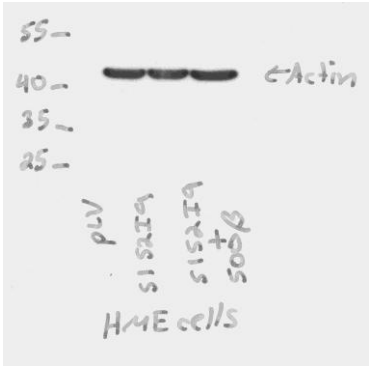

Supplement: Source Data for Figure 2A [file emboj2013203df2a.pdf]

Cheon *et al.* Source Data

Figure 4A

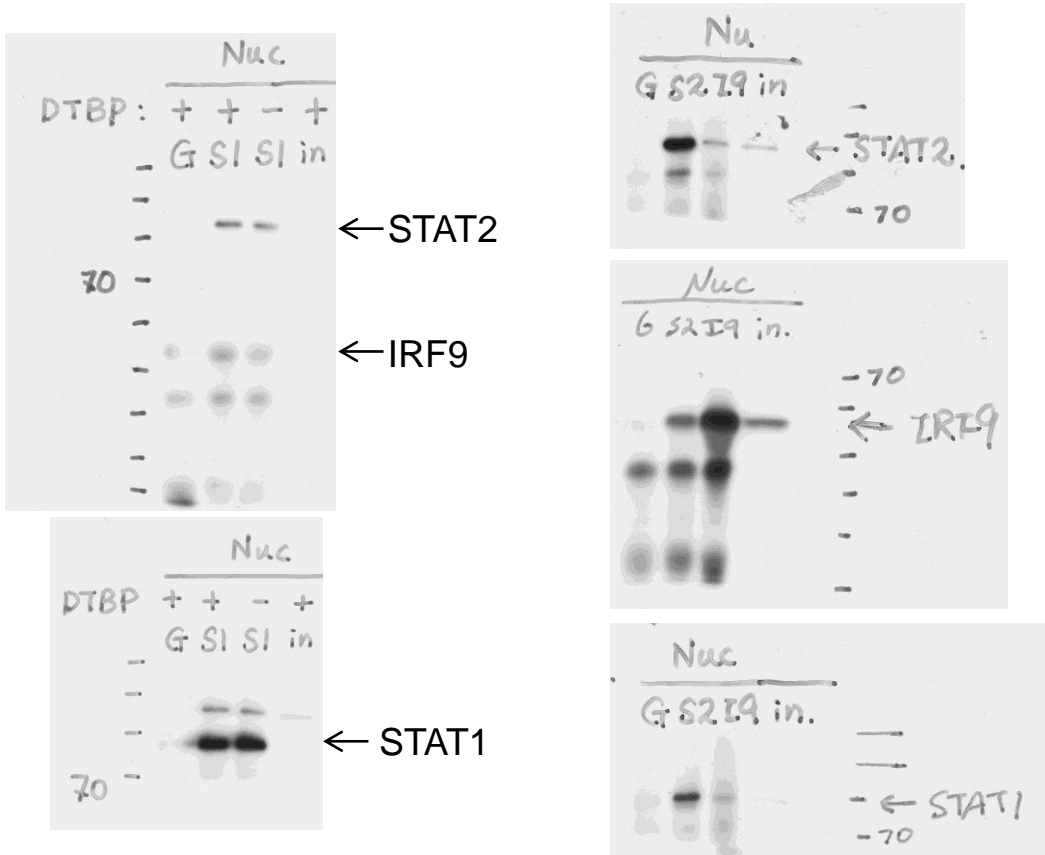

Supplement: Source Data for Figure 4A [file emboj2013203df4a.pdf]

Cheon *et al.* Source Data  
Figure 6A

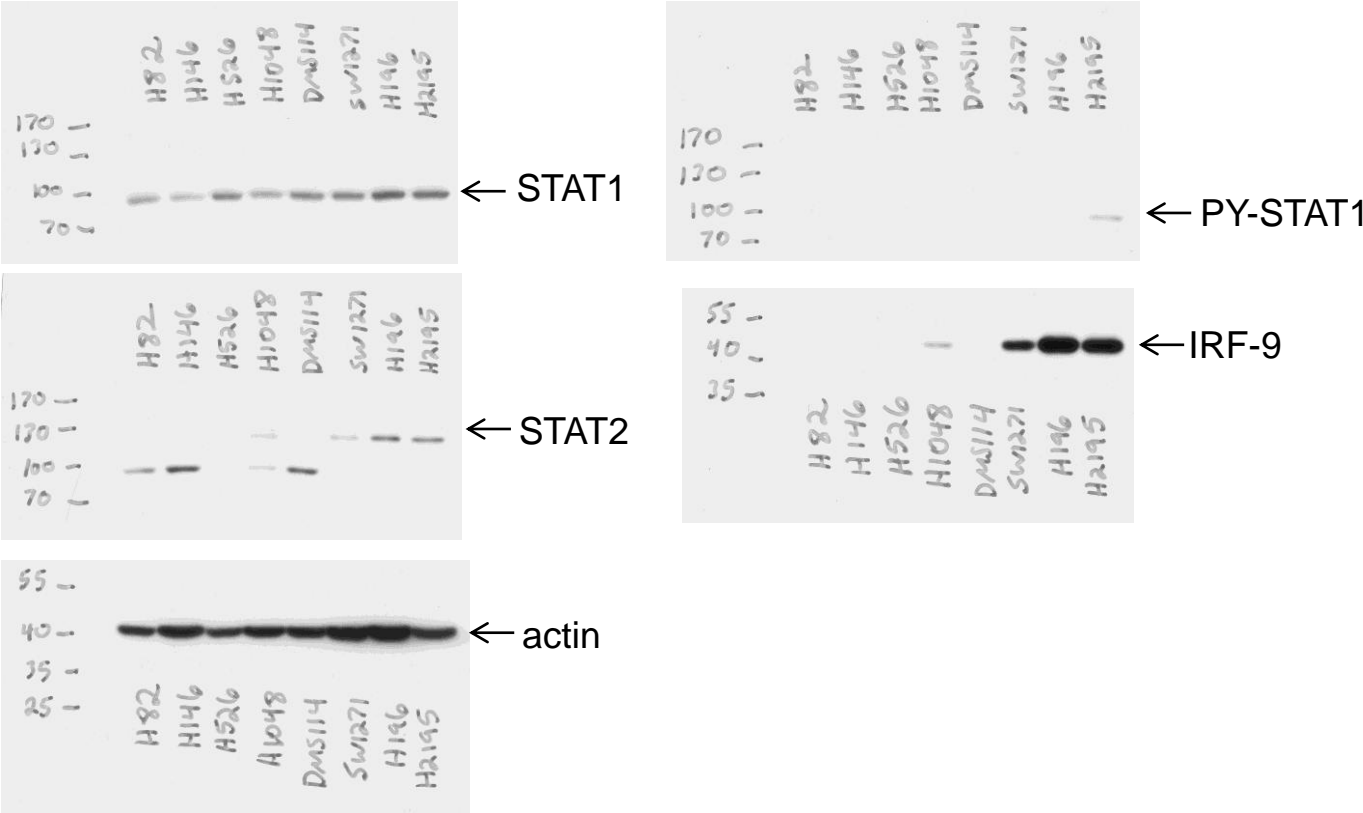

Supplement: Source Data for Figure 6A [file emboj2013203df6a.pdf]

Cheon *et al.* Source Data

Figure 6C

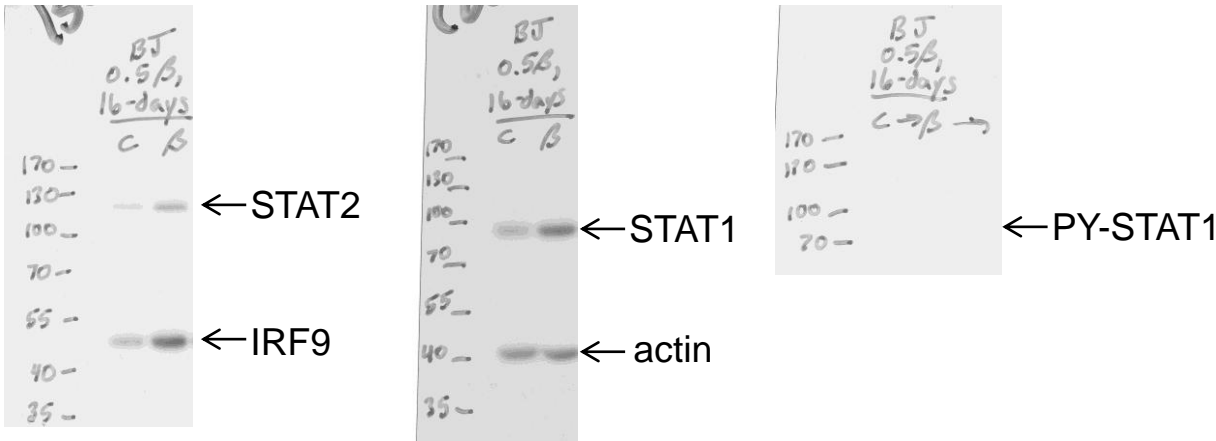

Supplement: Source Data for Figure 6C [file emboj2013203df6c.pdf]
